# Pennsylvania State Dental Society Clinics

**Published:** 1888-09

**Authors:** 


### PENNSYLVANIA STATE SOCIETY CLINICS.

Dr. William H. Trueman, Master of Clinics, made the following report :—

PHILADELPHIA, June 7th, 1888.

*To the President and Gentlemen of the Pennsylvania State Dental Society :—*

At the clinic held on Wednesday morning, June 6th, at Sibley's Dental Depot, Dr. F. J. Richards, of Williamsport, Pa., exhibited a device fitted to a Snow & Lewis automatic mallet, designed to facilitate its use in filling posterior approximal cavities. It forms a "back action" attachment to the mallet, and is as readily attached and removed as is the right angle attachment to the hand-piece of a dental engine. It seemed to be a practical suggestion.

Mr. E. E. Clark, representing the Electro Metallic Dental Plate Co., of Newark, N. J., exhibited specimens of their work. The plates shown were made of silver, heavily plated on each side with gold; they are produced by depositing the metal by electricity directly upon the plaster model, thereby securing a perfect fit. The model, it was claimed, is not injured during the process unless deep undercuts, or the shape of that portion of the plaster model covered by the plate renders its removal without fracture impossible. The plates are intended to serve as a base upon which may be moulded vulcanite, celluloid, etc.; and are not intended to have teeth soldered to them. It was stated that an extra deposit of metal could be made at any point desired to give additional strength, to form a rim, or to form projecting points or a roughened surface to afford secure attachment for the vulcanite or celluloid. The coating of gold on either side is sufficiently thick to protect the silver from injury during the process of vulcanizing. The metal seemed to be as solid and tenacious as metal that had been melted and rolled. The company propose to make the plates only, at a cost of $7.00 each. After the denture is finished; should the gold coating be injured at any point they undertake to renew it without additional cost, and without injury to the vulcanite or other attachment.

Dr. H. A. Parr, of New York, constructed a Removable Bridge piece for the upper jaw; attached anteriorly to the first bicuspid tooth and posteriorly to the second molar, filling the space from which the second bicuspid and first molar had been removed. The first bicuspid to which the anterior end of the bridge was attached was first, by means of corundum wheels in the dental engine, reduced in size and changed in shape to facilitate fitting over it a gold cap. A strip of coin gold was accurately fitted around it and the ends soldered to form a collar sufficiently wide so that while one edge, suitably shaped, fitted just under the free margin of the gum without, however, impinging unduly upon the periosteum, the other nearly reached the masticating surfaces of the opposing teeth when the mouth was closed. Over this end was now soldered, converting the collar into a cap, a piece of gold. Before soldering this piece to the collar it was shaped to represent the cusps of a natural bicuspid by laying it upon a piece of smooth wood, the ball end of a steel instrument handle placed upon it, the instrument being held at an angle of about forty-five degrees, when a quick blow with a wooden mallet upon the ball end neatly formed a cusp. The position of the instrument was now slightly changed and another like blow as quickly formed the second cusp. A little care in adjusting this to the collar produced a masticating surface corresponding almost as nearly to that of a natural bicuspid as though it had been swaged with a die. In a similar manner the cusps of a molar may be as readily and as quickly formed. The cap was now placed upon the tooth and firmly pressed home and the patient directed to close the teeth firmly; in doing so he crushed in the thin cap of gold covering the collar so as to make, as was designed, a perfect articulation with the opposing teeth. The cap was now removed and solder flowed inside to give the masticating surface sufficient stiffness and thickness to permit, should it be necessary in its final adjustment, the removal of a portion to accommodate its articulation with the opposing teeth without cutting entirely through. By this simple method Dr. Parr constructed quickly, and neatly, an accurately fitting bicuspid cap resembling very closely in form the natural tooth, all the work being done at the chair. He then, in like manner, constructed a cap for the molar tooth. After the caps were completed, to the posterior approximal surface of that fitting the bicuspid tooth was soldered, in line with the axis of the tooth, a short cylindrical tube, into which a pin was fitted and which was afterwards soldered to that end of the bridge. To the molar cap was soldered a box about one-twentieth of an inch wide and one-eighth of an inch long, and nearly as deep as the cap was wide; into this was fitted and soldered to the bridge a pin, corresponding in shape. In soldering the tube and box to their respective caps, care was taken to have them as nearly as possible in line, so that the pins, firmly attached to the bridge, would readily pass into them and fit tightly. When the arrangement was completed and the caps firmly cemented in place, the bridge, while perfectly firm when in place and not liable to displacement in mastication, was readily removed and replaced by the patient. The work was well done, notwithstanding the many inconveniences inseparably associated with clinical operations. Dr. Parr carefully explained each step of the process as he proceeded with his demonstration. The method of attachment was novel, and promises, where it is practical to use it, to add to the other advantages of bridge-work greater cleanliness and facility of repair.

Dr. E. Parmley Brown, of Flushing, N. Y., exhibited a number of specimens of his all porcelain bridge-work, and by means of plaster models explained his method of constructing and securing them in position. He also exhibited in the mouth of a patient a "bridged-in" bicuspid that had been in position over a year. The space to be supplied in this case was the first bicuspid of the upper jaw. Into this was fitted a bicuspid tooth (porcelain), having baked into it a platinum bar extending into a cavity made to receive it in the second bicuspid. The tooth was secured in place by first, with the electric mallet, packing gold to cover the floor of this cavity; the cervical margin was then neatly finished. The tooth was now placed in position and firmly held while gold was packed on either side and over the platinum bar, the contour of the natural tooth being fully restored. The operation was neatly done and looked well. The porcelain tooth pressing into the gum gave it a remarkably natural appearance, indeed; a close inspection was needed to detect it. Dr. Brown prefers that the teeth should press upon the gum firmly. The smooth vitrified surface of porcelain does not favor the accumulation of matter to so great a degree as does a metallic surface. He finds the work more cleanly when so arranged.

The clinic on Thursday morning, June 7th, was held at Justi's Hall.

Dr. E. C. Kirk implanted the right superior central for a patient for whom he had recently successfully implanted a left superior lateral; the latter had become quite firm, and in appearance and usefulness was all that could be desired. His method of performing the operation did not differ materially from that usually pursued; first, opening from the apex of the root and thoroughly cleansing the pulp-canal and the pulp-chamber, then filling the larger portion of this space with gutta percha, and finally sealing the opening in the apex with a neatly finished filling of gold. Before commencing to operate upon the patient he injected hypodermically a solution of cocaine into the gum over the point where the artificial socket was to be made, and waited a few minutes until the cocaine had had the desired effect. He advised caution in the use of cocaine; one-eighth of a grain being the maximum amount he thought prudent to use; in some cases less will answer. The effect of the drug is not always prompt, a few minutes being required for its absorption. He would rather wait a little longer than risk using a larger amount. In this case, probably five minutes elapsed before the sensitiveness of the parts was sufficiently reduced to permit the operation to proceed. He also urged the importance of thoroughly sterilizing the tooth to be implanted, and all the instruments to be used, and keeping them in that condition throughout the operation. For this purpose he used two solutions of mercuric bichloride, a weak one in which the tooth was placed, and a stronger one in which the instruments were kept. These solutions were kept at a temperature of about 98° by means of the self-regulating apparatus designed by Dr. Kirk for this purpose, and on sale at the dental depots. He recommended that the solutions be made with distilled water in all cases, otherwise a slight precipitation of the bichloride of mercury was possible, which would impair their efficiency.

The operation was successfully performed. The patient gave no evidence of suffering, and stated that the operation had not been really painful.

Dr. A. G. Bennett, of Philadelphia, by means of plaster models demonstrated a method of securing accurate impressions in difficult cases. In cases where an isolated tooth is required, and the space is narrow, or from other causes there is difficulty in securing a correct impression of the space, he suggests; first, fitting into it the artificial tooth that is to be used, in the position it is to occupy, then taking the impression while it is in place, letting it form part of the impression.

In cases where the teeth lean in excessively, he suggests building a wall, quite wide at its base, of yellow wax, upon the impression cup sufficiently high to nearly cut through the plaster (he prefers plaster for taking impressions in these cases), when the cup is pressed in place. After the plaster of the impression has hardened, the cup is removed, leaving the impression in place in the mouth. The soft wax is readily removed, and there is then no difficulty in fracturing the plaster and removing the impression in sections. The wall of wax is placed so as to divide the impression into sections convenient for removal.

He also illustrated by models a method of "cap and groove anchorage" for bridges and crowns. This is especially applicable to the bicuspid teeth, especially to those which have a cavity of decay upon one or both approximal surfaces. He first grinds enough from the palatal or lingual surface to permit the adaptation of a neatly fitting collar of gold encircling the tooth, except upon its buccal surface. He also cuts away the inner cusp sufficient to admit the thickness of the cap without interfering with the articulation of the opposing teeth, and forms across the masticating surface a slight groove in which the edge of the cap may rest. This usually converts the approximal cavities into open grooves. Should it not do so, they are so made by extending them through to the masticating surface. A gold or platinum collar is now fitted covering the lingual surface and both approximal surfaces of the tooth. This is firmly held in place while, with a pair of round nose plyers, the beaks of which are longer than usual, and so shaped that they are nearly parallel to each other when grasping the tooth, the collar is bent in on each side to fill the grooves. A little care is required in doing this to draw the collar closely to the tooth; if unskillfully done the collar will be made too large on the lingual side, and will not "hug" the tooth sufficiently close. A piece of gold is now fitted to cover the inner cusp, extending over the masticating surface to the groove made for its reception, and soldered to the collar. It is now placed on the tooth and the edges burnished closely to the tooth at all points. A staple of stiff half round gold wire is fitted over it so as to slide into the grooves on either side, in such a manner that the convexity of the wire presents on the masticating surface. He now carefully removes the cap with the staple in position and solders them together, using sufficient solder to well fill the grooves; this not only stiffens the cap, but permits a neater finish on the approximal surfaces. The cap is secured to the tooth with zinc phosphate cement. The doctor suggests this form of cap, not only as a support for bridge-work, but also as a ready and effective method of repairing bicuspid teeth badly decayed on their approximal surfaces.

Dr. Bennett also exhibited a method of using the "Bing" style of teeth in constructing bridge-work. These teeth are mostly bicuspids, and are made to closely resemble, in shape, the natural teeth; the pins are on the approximal surface of each side. In mounting them for bridge-work he first grinds away the bulging portion of the lingual surface, then fits a collar of suitable width extending around the lingual surface, and sufficiently far on each approximal surface to embrace the pins which pass through holes made to accommodate them; for convenience this collar is made in two pieces, joining about the middle of the lingual surface. When the collar is fitted and in place, the pins are riveted over it by splitting them with a sharp instrument. The tooth is now invested, and the pins of each side and the joining at the centre of the collar securely soldered. After all the teeth to be used have been thus mounted they are arranged in place, the ends of the collars being filed thin, if need be, to permit the teeth to approximate, invested and soldered together. After the bridge is finished, with the electric mallet he neatly packs gold into any spaces that may exist between the teeth. This promises to be a useful suggestion. It is seemingly strong, there is no gold exposed, it is cleanly, and more readily constructed than the usual method of using thin porcelain facings and forming the lingual and masticating surfaces with metal.

Dr. H. C. Register, of Philadelphia, exhibited his new dental engine. This has many points of excellence that cannot, however, well be explained without drawings.

The Detroit Electric Motor for dental purposes was shown in connection with a battery designed by the Partz Electric Battery Co., of Philadelphia, especially for running motors.

Dr. W. B. Miller, of Altoona, Pa., exhibited a set of loop or band matrices which he has recently designed.

Dr. Carroll, of Meadville, Pa., successfully cast a partial upper plate of his alluminium alloy, and demonstrated the use of his apparatus for that purpose.

